# Risk factors for surgical site infection in patients undergoing obstetrics and gynecology surgeries: A meta-analysis of observational studies

**DOI:** 10.1371/journal.pone.0296193

**Published:** 2024-03-06

**Authors:** Zhan Yang, Dong Wang, Min Yang, Jianjun Deng, Yin Liu

**Affiliations:** 1 Medical Department, West China Second University Hospital, Sichuan University, Chengdu, Sichuan, China; 2 Key Laboratory of Birth Defects and Related Diseases of Women and Children (Sichuan University), Ministry of Education, Chengdu, Sichuan, China; 3 Nosocomial Infection Management Department, West China Second University Hospital, Sichuan University, Chengdu, Sichuan, China; 4 Department of Laboratory Medicine, Chengdu Jinniu District Center for Disease Prevention and Control, Chengdu, Sichuan, China; Tehran University of Medical Sciences, ISLAMIC REPUBLIC OF IRAN

## Abstract

**Objective:**

The aim of this study was to identify the risk factors for surgical site infection (SSI) in patients undergoing obstetrics and gynecology surgeries through meta-analysis.

**Methods:**

Relevant original studies published from January 1945 to May 2023 were searched the CBM, PubMed, Embase, WOS, CNKI, Wanfang, vip, and Cochrane Library databases. Studies eligible were evaluated by two investigators following Newcastle-Ottawa Scale(NOS) criteria. Review Manager 5.3 software was used to analyse the combined effect sizes and test for heterogeneity, and Stata 14.0 software’s Begg’s Test and Egger’s Test were used to test for bias.

**Results:**

13 case-control articles, including 860 cases in the case group and 13574 cases in the control group, met the inclusion criteria. Eventually, Our meta-analysis showed that SSI in patients undergoing obstetrics and gynecology surgeries was correlated with body mass index (BMI)≥24 (OR = 2.66; *P* < 0.0001), malignant lesions (OR = 4.65; *P* < 0.0001), operating time≥60min (OR = 2.58; *P* < 0.0001), intraoperative bleeding≥300ml (OR = 2.54; *P* < 0.0001), retained urinary catheter (OR = 4.45; *P* < 0.0001), and vaginal digital examination≥3times (OR = 2.52; *P* < 0.0001).

**Conclusion:**

In this study, BMI≥24, intraoperative bleeding≥300ml, malignant lesions, operating time≥60min, retained urinary catheter, and vaginal digital examination≥3times were considered as independent risk factors for SSI in obstetrics and gynecology surgery. It is recommended that scholars be rigorous in designing the experimental process when conducting case-control or experimental studies in order to improve the quality of the study. Controlling patients’ weight before obstetrical and gynecological surgery, shortening the operation time intraoperatively, and strictly controlling the indications of vaginal digital examination and retained urinary catheter can effectively reduce the incidence of SSI.

## Introduction

Hysterectomy is one of the three most commonly performed procedures in gynecology [1], while cesarean section is the most commonly performed procedure in obstetrics and constitutes approximately 40% of all deliveries in China [2, 3]. Incisions in obstetrics and gynecology surgery are often placed on the skin, vulva, vagina, and other places where a large number of microorganisms exist. These incisions are extremely susceptible to infection. At the same time, infection is associated with increased hospitalization time and elevated health care costs [4]. Of the infections, SSI, which affects surgical therapeutic outcomes, is the most prevalent hospital-based infection [4]. In China, the incidence of SSI after obstetric and gynecologic surgery is 4.62% [5]. The incidence of SSI after hysterectomy ranged from 2.3% to 8.1% [6–8], and the incidence of SSI after cesarean section ranged from 3% to 16% [9–11]. However, the risk factors for SSI are complex and difficult to identify. Current findings on risk factors in the literature are often limited by small sample sizes and weak statistical power. The aim of this study was to provide an evidence-based theoretical basis as well as scientific recommendations for the prevention of surgical site infections in obstetrics and gynecological surgery by combining and analyzing the outcome data from several related publications.

## Research methodology

### Search strategy

Eight databases were searched in CBM, Wanfang, CNKI, VIP, Pubmed, WOS, Cochrane Library, and Embase according to the search strategy (inclusion date:May 12, 2023). The search terms will follow the standard PICO guideline (population, intervention, comparator, outcome) and were developed according to disease category (gynecological surgery or obstetric surgery) and study purpose (surgical site infection). The search formula was developed by combining free words with subject terms, and the Medical Subject Headings (MeSH) terms were searched in the Pubmed database [12, 13].

### Inclusion criteria and exclusion criteria

The selection of studies was first performed on the basis of titles and abstracts. Then two authors (Yin Liu and Dong Wang) independently screened the full text of the identified papers using the following inclusion criteria: (1) studies must meet the National Healthcare Safety Network’s definition of SSI: a wound infection that occurs within 30 days of an operative procedure or within a year if an implant is left in place and the infection is thought to be secondary to surgery [14]; (2) Statistics must be included in the multi-factor analysis after univariate analysis of significant indicators; (3) studies providing effect estimates of the relative risks (RRs) or odds ratios (ORs) with 95% confidence intervals (CIs); (4) case-control or cohort studies.

Review articles, conference abstracts, animal experiments, meta-analyses, and studies with insufficient or overlapping data were excluded from this study. Mediolateral episiotomy, vulval surgeries, repair of perineal tears, hysteroscopic surgery and cervical surgery, were also excluded at the same time.

### Quality assessment

The quality of all the included studies was evaluated by NOS based on the three modules: the selection of case group and control group (0–4 points), inter-comparability of groups (0–2 points), exposure and outcomes (0–3 points), with a maximum score of 9. The studies with NOS scores ≥ 6 were considered relatively higher quality [15].

### Data extraction

For all eligible studies, the following variables were extracted by two authors (Yin Liu and Dong Wang): (1) the first author’s name; (2) publication year; (3) year of the study; (4) country; (5) risk factors; (6) surgical types; (7) study type; (8) statistical methods; (9) numbers of cases and controls; and (10) estimates of odds ratios (ORs) or relative risks (RRs). Any disagreement was settled by the third reviewer (Zhan Yang).

### Statistical analysis

All the statistical analyses were performed with RevMan 5.3 (The Nordic Cochrane Centre, Copenhagen, Denmark) and Stata 14.0 (Stata Corporation, College Station, TX). For all risk factors in our study, adjusted ORs and 95% CIs were extracted from the original studies. A two-tail *P* value less than 0.05 was considered significant. Heterogeneity was tested by the Q-test (with significance set at *P* < 0.10) and *I*^*2*^ statistics (with *I*^*2*^ > 50% implying heterogeneity). In the case of significant heterogeneity, we use sensitivity analysis to recognize the potential contribution of each study to the heterogeneity by removing one study at a time. If heterogeneity still existed, random-effects models were used; otherwise, fixed-effects models were used.The outcomes of the meta-analysis were summarized by the forest plot.

## Result

### Study selection and evaluation

**Fig 1:**
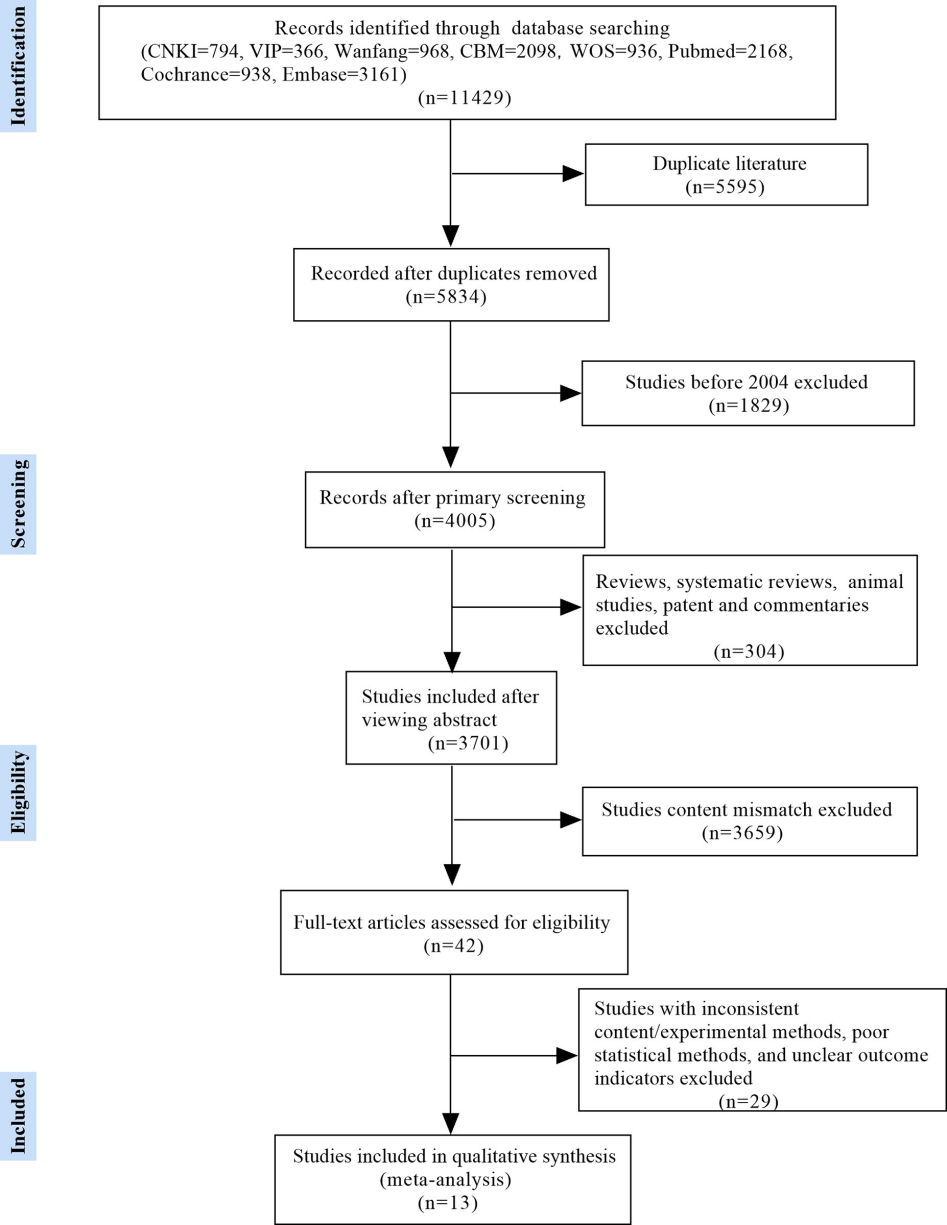
The procedure of literature selection.

A total of 11429 potentially eligible studies were identified by the initial database search, of which 3701 were included after excluding those published before 2004, duplicates, reviews, animal studies, patents, and commentaries. After screening titles and abstracts, 42 articles were included. After reading the full article carefully, 13 retrospective case control studies that were published between 2017 and 2023 were included (Fig 1). The outcomes of the NOS score for these 13 articles were as follows: One study scored 8; five studies scored 7; and seven studies scored 6. Literature with an assessment score of 5 or more was included in the meta-analysis, and all 13 papers were included in the meta-analysis. Detailed information about those 13 studies is presented in Table 1.

**Table 1:** Detail information about 13 studies.

Author	Publication year	Study type	Case group	Control group	Comparison source	Risk factors
Arakaki, Y [16]	2019	Case-control study	10	97	Hospital	2.7
Ye, Q [17]	2019	Case-control study	149	1071	Hospital	2.2、5.3、6
Ma, X J [18]	2017	Case-control study	47	1299	Hospital	2.5、3.2、4.4、6
Wei, F F [19]	2020	Case-control study	16	182	Hospital	2.1、3.1、4.5、5.1、6
Xu, C W [20]	2020	Case-control study	75	953	Hospital	2.4、4.1、5.3、7.2、8.3
Weng, Y R [21]	2018	Case-control study	51	828	Hospital	1.2、4.2
Xie, Z Y [22]	2017	Case-control study	54	1042	Hospital	1.1、2.1、4.2、7.2、8.1
Zhang, G [23]	2021	Case-control study	94	884	Hospital	4.5
Zhu, Z Q [24]	2019	Case-control study	64	1422	Hospital	4.1、5.2、8.2
Wang, J Y [25]	2019	Case-control study	27	3533	Hospital	1.3
Abdel Jalil, M H [26]	2017	Case-control study	124	737	Hospital	2.7
Li, L [27]	2021	Case-control study	48	158	Hospital	2.3、4.4、5.2、7.1
Xie, Z Y [28]	2019	Case-control study	101	1368	Hospital	3.2、4.5

Risk factors: 1.1 Hemoglobin<90g/L, 1.2 Hemoglobin<100g/L, 1.3 Anemia; 2.1 BMI>24, 2.2 BMI>25, 2.3 BMI≥24, 2.4 BMI≥25, 2.5 BMI≥28, 2.6 BMI≥30, 2.7 BMI≥36; 3.1 Malignant lesions, 3.2 Malignant tumors; 4.1 Operation time>60min, 4.2 Operation time≥60min, 4.3 Operation time≥90min, 4.4 Operation time≥95min, 4.5 Operation time≥180min; 5.1 Intraoperative bleeding≥300ml, 5.2 Intraoperative bleeding≥400ml, 5.3 Intraoperative bleeding>500ml; 6 Diabetes; 7.1 Retained urinary catheter≥24h, 7.2 Retained urinary catheter; 8.1 Vaginal digital examination>3, 8.2 Vaginal digital examination≥3, 8.3 Vaginal digital examination≥5.

### Meta-analysis

#### Combined analysis of effect sizes

A fixed model was selected to analyze anemia, BMI, malignant lesions, surgery time, intraoperative bleeding, diabetes, retained urinary catheter, and vaginal digital examination, and the results are shown sequentially in Figs 2–9.

**Fig 2:**
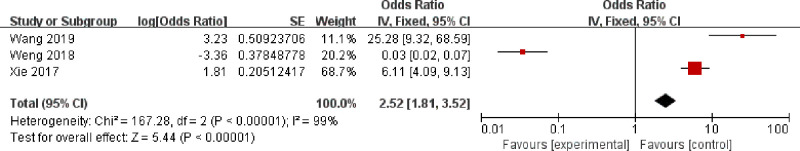
Anemia.

**Fig 3:**
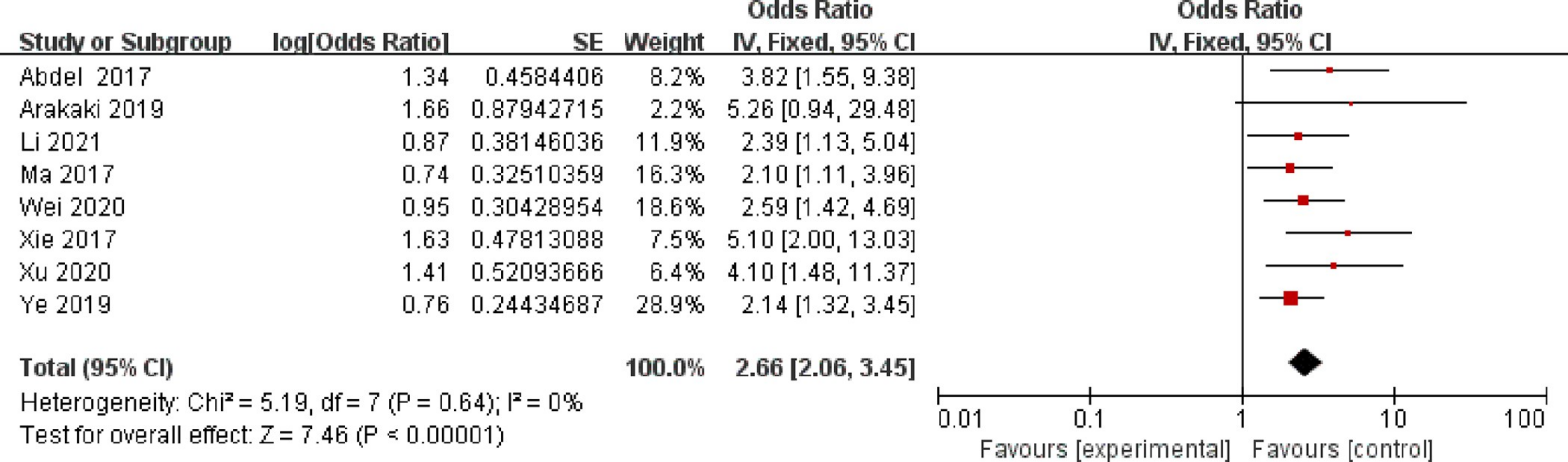
BMI.

**Fig 4:**
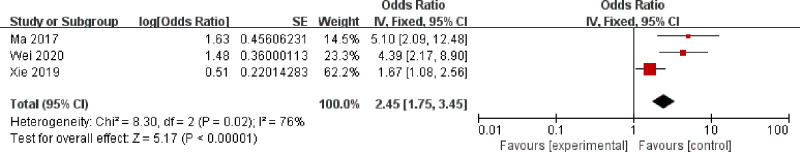
Malignant lesions.

**Fig 5:**
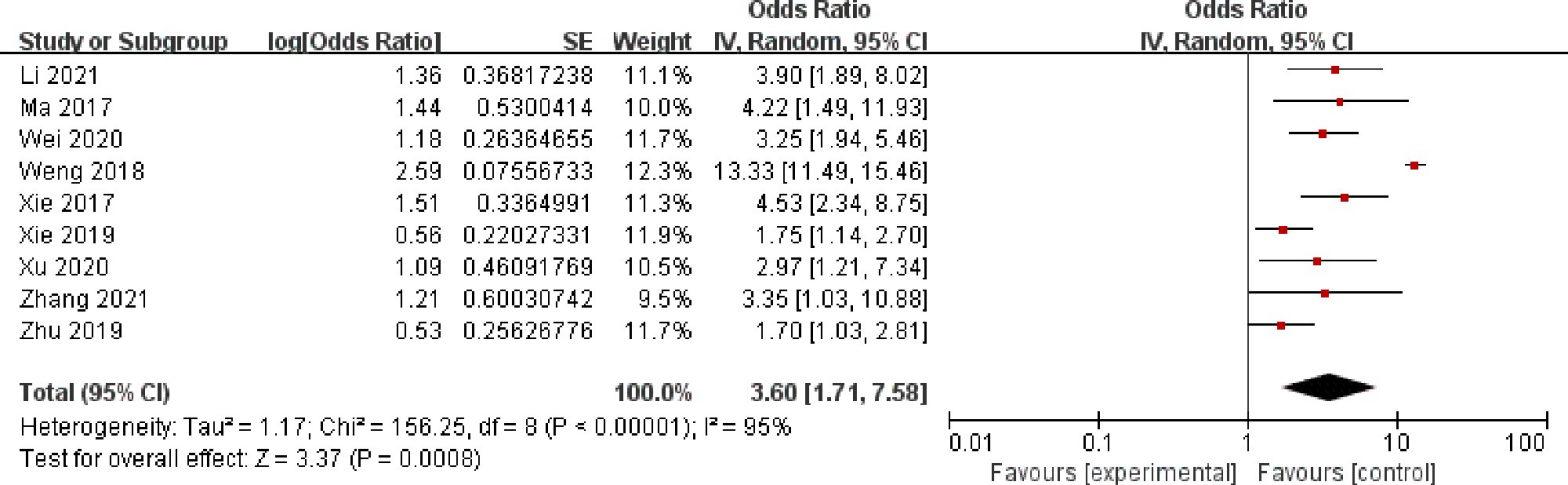
Surgery time.

**Fig 6:**
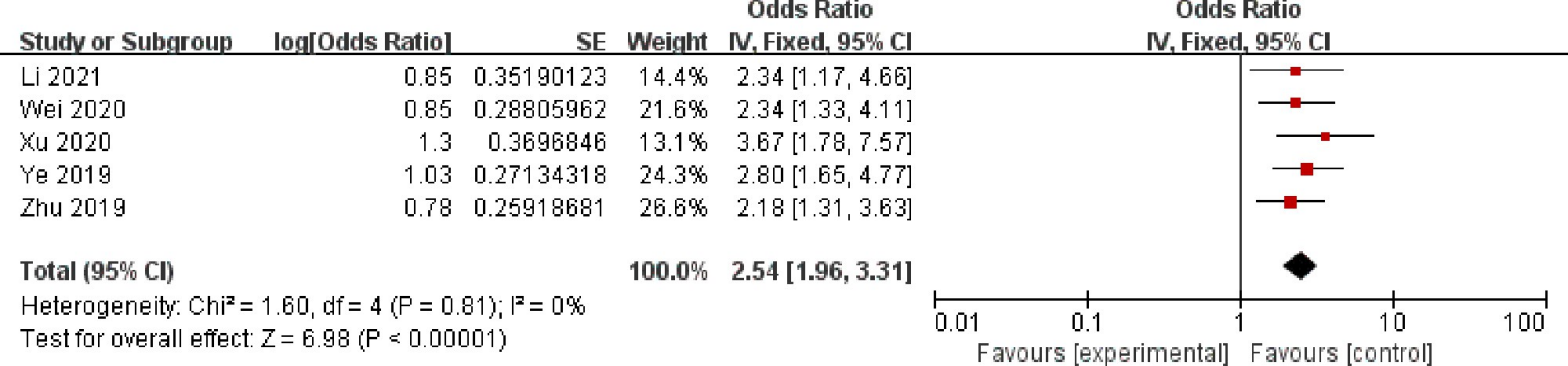
Intraoperative bleeding.

**Fig 7:**
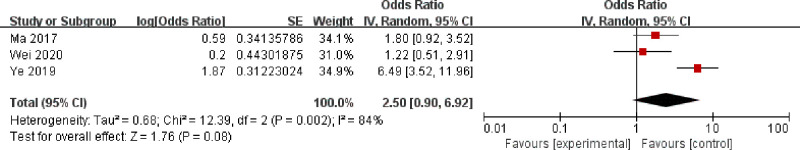
Diabetes.

**Fig 8:**
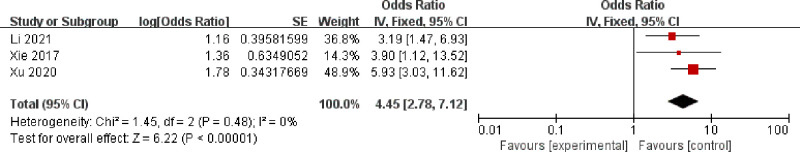
Retained urinary catheter.

**Fig 9:**
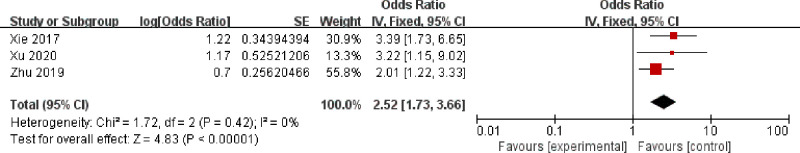
Vaginal digital examination.

As can be seen from Figs 2–9: BMI, intraoperative bleeding, retained urinary catheter, and vaginal digital examination were independent risk factors for surgical site infection after obstetrical and gynecological surgery (*p* < 0.05); there was significant heterogeneity in the results of the surgery time, malignant lesion, diabetes and anemia *(I*^*2*^ > 50%, *p* < 0.1), and sensitivity analysis needs to be continued.

#### Sensitivity analysis

**Fig 10:**
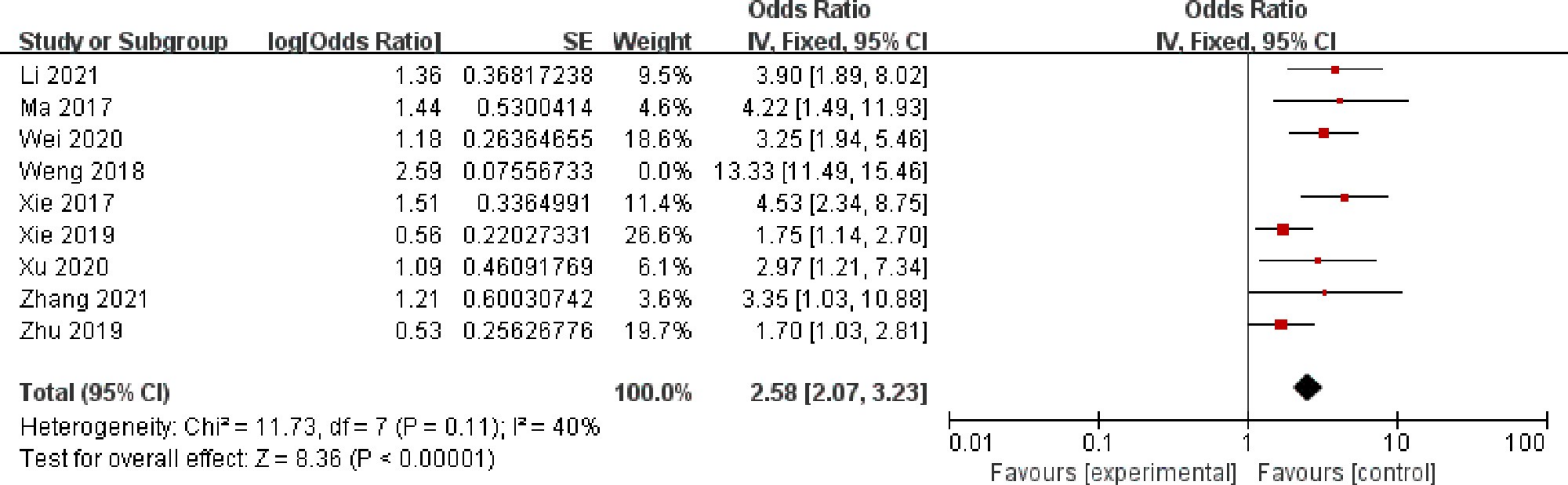
Surgical duration-sensitivity analysis.

**Fig 11:**
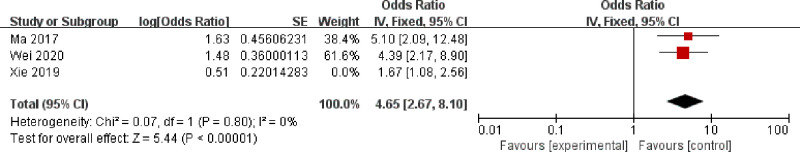
Malignant lesions-sensitivity analysis.

**Fig 12:**
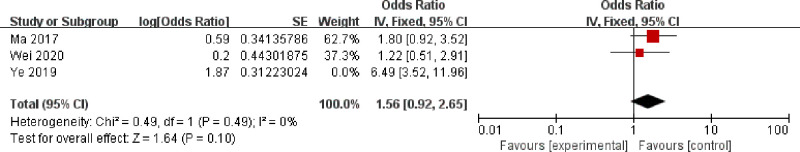
Diabetes-sensitivity analysis.

**Fig 13:**
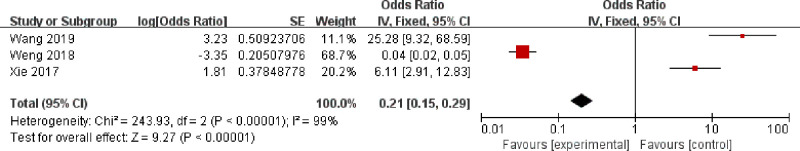
Anemia-sensitivity analysis.

The heterogeneity of surgery time(Fig 10) was significantly reduced after removing Weng YR 2018. Xie ZY 2019 in the malignant lesion study(Fig 11) and Ye Q 2019 in the diabetes study(Fig 12) were the main causes of heterogeneity, and the meta-analysis was performed again after removing the literature that caused heterogeneity, which yielded: surgery time and malignant lesions were independent risk factors for SSI in obstetrical and gynecological surgery (*p* < 0.05). In studies involving anemia(Fig 13), the result was *I*^*2*^ ≥ 99%, regardless of which study was deleted.

#### Bias test

**Fig 14:**
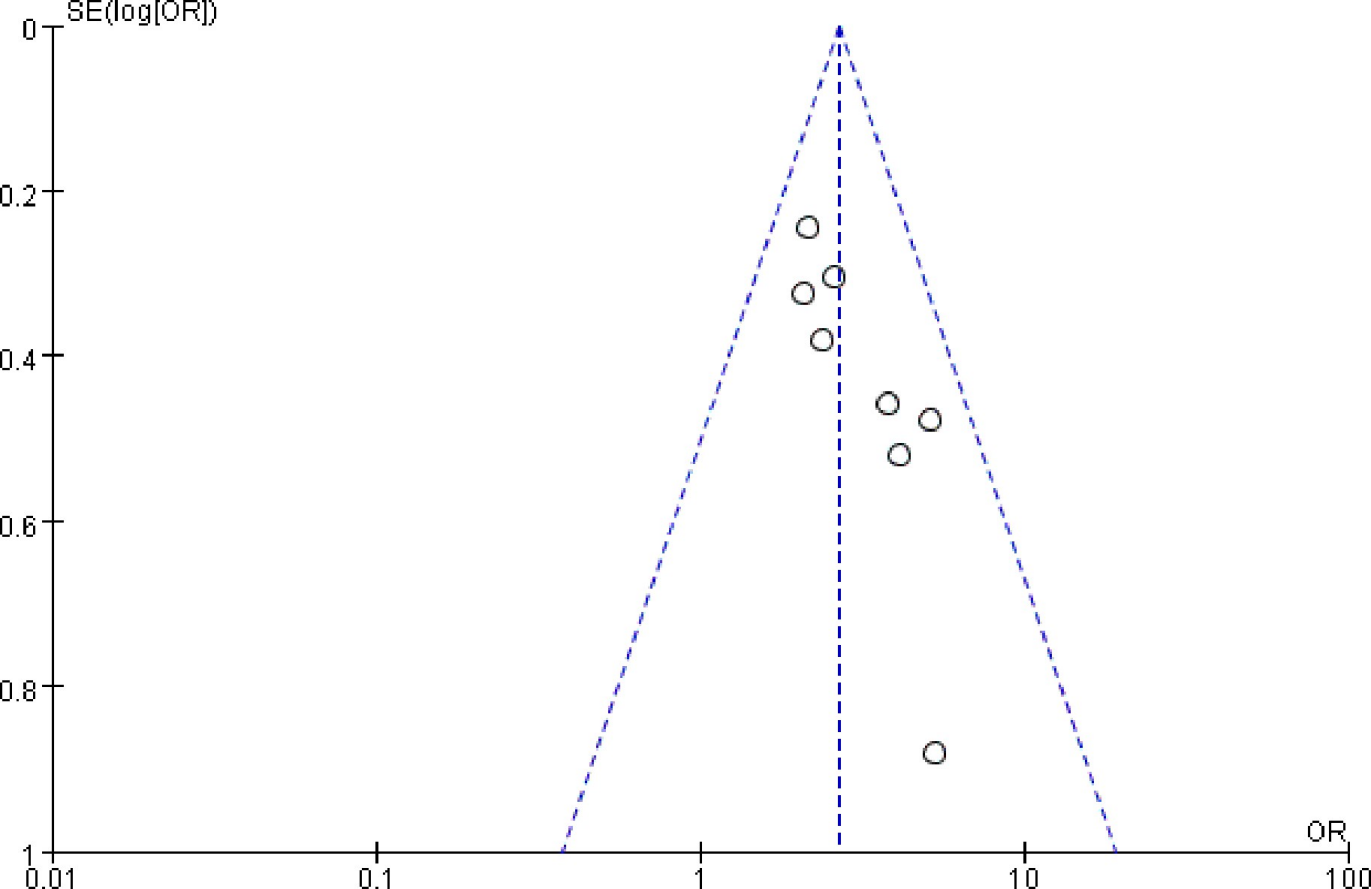
BMI funnel chart.

**Fig 15:**
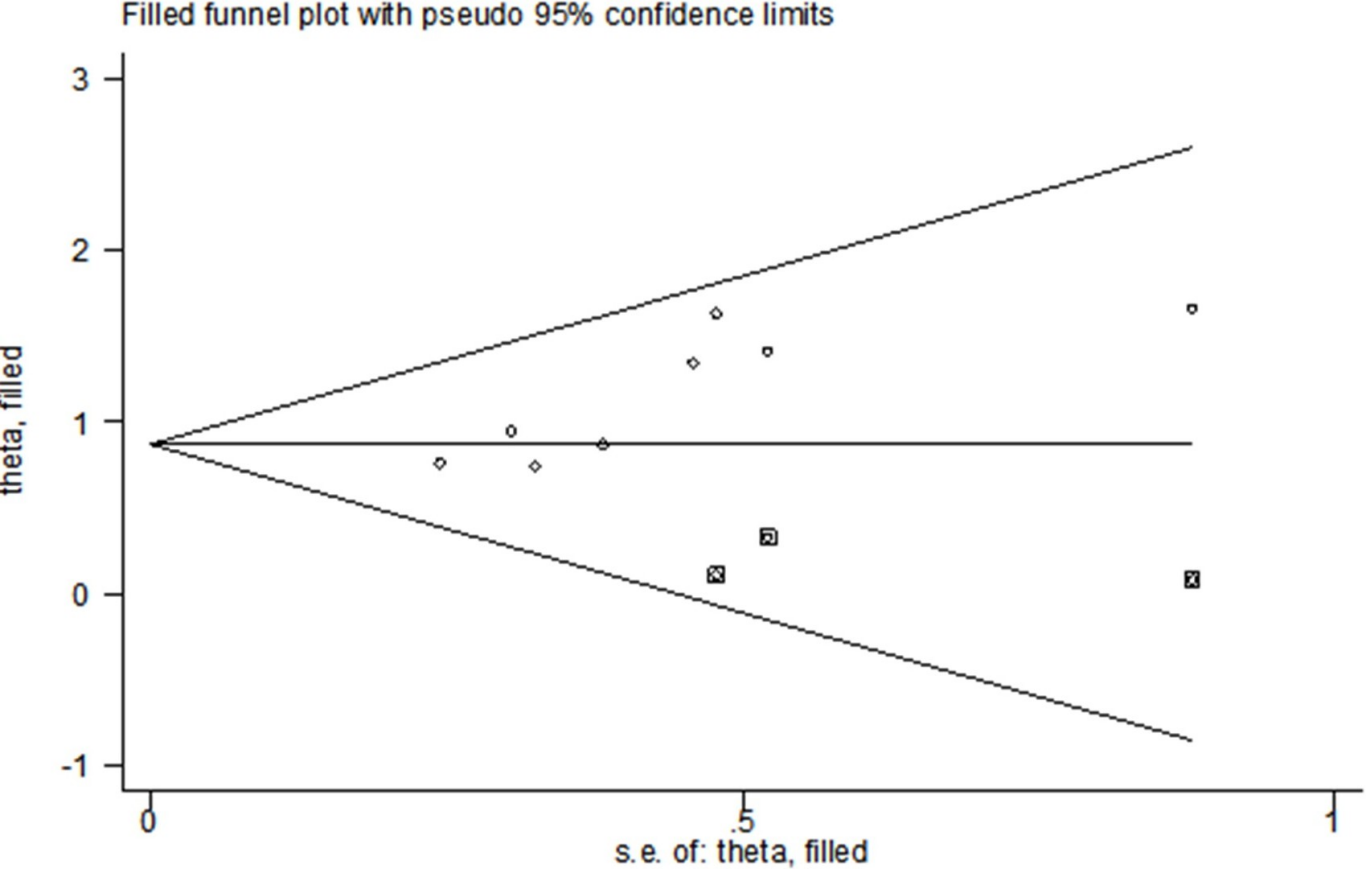
Subtractive complementation.

Separate tests of bias for BMI, operative time, intraoperative bleeding, retained urinary catheter, vaginal digital examination, and malignant lesions yielded: operative time (*t* = 1.99, *P* > |*t*| = 0.094 > 0.05), intraoperative bleeding (*t* = 1.19, *P* > *|t|* = 0.320 > 0.05), retained urinary catheter (*t* = -0.47, *P* > |*t*| = 0.721 > 0.05), vaginal digital examination (*t* = 1.13, *P* > *|t*| = 0.461 > 0.05), and malignant lesions (*z* = 1.00, *Pr* > *|z|* = 0.317 > 0.05) were not publication biased. There was a mild publication bias in the BMI funnel plot (Fig 14) (*t* = 3.72, *P* > |*t|* = 0.01 < 0.05). The asymmetric funnel plot was processed by the cut-and-patch method, and the symmetry of the funnel plot could be ensured and publication bias eliminated by the three points of the square in Fig 15, indicating the need to include future effect size studies with results close to those of Arakaki Y 2019, Xu CW 2020, and Xie ZY 2017 (Fig 15).

## Discussion

SSI is one of the most common complications after obstetric and gynecologic surgery [29, 30]. Previous studies have identified many risk factors, including BMI, operating time, vaginal digital examination, intraoperative bleeding, diabetes, obesity, and malignant lesions [31–36]. However, these studies usually focused on only some of the risk factors and lacked a comprehensive quantitative summary of all the risk factors for SSI in obstetric and gynecologic surgery. A total of 13 articles were included in this study, including 860 cases in the case group and 13574 cases in the control group. Eventually, our meta-analysis showed that BMI≥24 (OR = 2.66; *P* < 0.0001), malignant lesions (OR = 4.65; *P* < 0.0001), operating time≥60min (OR = 2.58; *P* < 0.0001), intraoperative bleeding≥300ml (OR = 2.54; *P* < 0.0001), retained urinary catheter (OR = 4.45; *P* < 0.0001), and vaginal digital examination≥3times (OR = 2.52; *P* < 0.0001) were independent risk factors for SSIs in obstetrics and gynecology surgery.

The greatest risk factor for SSI in obstetric and gynecologic surgery is malignant lesions (OR = 4.65), which increase the likelihood of SSI by 365%. Malignant lesions have long been recognized as a major source of postoperative infections [37, 38]. The immune system is generally compromised in patients with malignant tumors [39]. This impairment in the primary immune function directly results from the tumor’s pervasive influence on the natural defense mechanisms [40]. In addition, standard therapeutic interventions for tumors, including surgery, chemotherapy, and radiation therapy, also lead to weakened immune function [41]. The second largest risk factor is retained urinary catheter (OR = 4.45), which has a 355% increased likelihood of SSI. Retaining a urinary catheter is, on the one hand, an invasive operation itself, and on the other hand, the friction of the tube in the urethra can cause inflammation [42–44].

The study by Li Jing et al. also concluded that BMI, operating time, vaginal digital examination, and intraoperative bleeding were risk factors for SSI in obstetric and gynecologic surgery, but that article did not give specific values for BMI, operating time, or intraoperative bleeding. The results of this study showed that anemia and diabetes mellitus were not risk factors for SSI in obstetric and gynecologic surgery, which is inconsistent with the findings of Li Runrong et al. The possible reason is that BMI and diabetes may be interlinked, and obesity-induced insulin resistance is one of the major sources of type 2 diabetes [45–48]. Therefore, most of the studies selected only one of the two factors for analysis. Only three of the 13 articles included in this study analyzed diabetes, and more data support is needed for a more scientific conclusion.

To ensure the reliability of the conclusions of the analysis and the homogeneity of the study outcomes, three aspects of the included literature, namely, clinical research direction, experimental design methodology, and statistics [49], were strictly controlled during the literature screening process in this study [50, 51]. In terms of clinical study orientation, confounding factors such as perineal surgeries were excluded from this study because the female lower genital tract is connected to the outside world and hosts a variety of colonizing bacteria, mycoplasma, chlamydia, and pseudofilamentous yeasts [52]. Although surgical sites were excluded, there are many different types of obstetric and gynecologic surgery, including total laparoscopic hysterectomy, abdominal hysterectomy, and total vaginal hysterectomy, etc. The type of surgery may also be an influencing factor for SSI [53], and this point was not explored in this article.

## Conclusion

In this study, BMI≥24, intraoperative bleeding≥300ml, malignant lesions, operating time≥60min, retained urinary catheter, and vaginal digital examination≥3times were considered as independent risk factors for SSI in obstetrics and gynecology surgery.According to the results of this study, in order to reduce the incidence of SSI in obstetrics and gynecology surgery, the medical staff should carry out a comprehensive assessment of the patient before the surgery and formulate a reasonable surgical program. In patients undergoing planned obstetric and gynecologic surgery, weight management should be done. Rational prophylactic use of antimicrobials before performing surgery for patients with malignant lesions. Surgical methods, surgical instruments, and experienced medical staff should be rationally selected to minimize the surgical incision in order to shorten the operation time and reduce intraoperative bleeding. When estimating the progress of labor, focus on the observation of the mother’s condition, such as the contraction of the uterus, the heartbeat of the fetus, etc., and reduce the number of vaginal digital examinations. Post-operative observation of the patient should be strengthened, and the catheter should be removed as early as possible.

## Supporting information

S1 Checklist PRISMA 2009 checklist. (DOCX)

S1 File Search strategy. (DOCX)
