# Supplementary material for: Risk factors for surgical site infection in patients undergoing obstetrics and gynecology surgeries: A meta-analysis of observational studies
Source: PLoS One. 2024 Mar 6;19(3):e0296193. doi: 10.1371/journal.pone.0296193 (PMC10917295; doi:10.1371/journal.pone.0296193)
Supplement: S1 File — (DOCX) [file pone.0296193.s002.docx]

**P：Gynecological surgery**

**subject：**

Gynecologic Surgical Procedures

**Mesh：**

Procedures, Gynecologic Surgical

Surgical Procedure, Gynecologic

Surgery, Gynecological

Gynecological Surgeries

Gynecological Surgery

Surgeries, Gynecological

Gynecologic Surgical Procedure

Surgical Procedures, Gynecologic

Gynecological Surgical Procedure

Gynecological Surgical Procedures

Procedure, Gynecological Surgical

Procedures, Gynecological Surgical

Surgical Procedure, Gynecological

Surgical Procedures, Gynecological

Procedure, Gynecologic Surgical

Gynecologic Surgery

Gynecologic Surgeries

Surgeries, Gynecologic

Surgery, Gynecologic

**P：Obstetric surgery**

**Subject：**

Obstetric Surgical Procedures

**Mesh：**

Obstetric Surgical Procedure

Procedure, Obstetric Surgical

Obstetrical Surgical Procedures

Obstetrical Surgical Procedure

Procedure, Obstetrical Surgical

Procedures, Obstetrical Surgical

Surgical Procedure, Obstetrical

Surgical Procedures, Obstetrical

Surgical Procedure, Obstetric

Surgical Procedures, Obstetric

Surgery, Obstetric

Obstetric Surgeries

Obstetric Surgery

Surgeries, Obstetric

Obstetrical Surgery

Obstetrical Surgeries

Surgeries, Obstetrical

Surgery, Obstetrical

Procedures, Obstetric Surgical

**I：**Surgical Wound Infection

**subject：**

Surgical Wound Infection

**Mesh：**

Infections, Surgical Wound

Surgical Wound Infections

Wound Infections, Surgical

Infection, Surgical Wound

Surgical Site Infection

Infection, Surgical Site

Infections, Surgical Site

Surgical Site Infections

Wound Infection, Postoperative

Wound Infection, Surgical

Infection, Postoperative Wound

Infections, Postoperative Wound

Postoperative Wound Infections

Wound Infections, Postoperative

Postoperative Wound Infection

**1.PubMed**

**time：2023/5/10 21:37:30**

**quantities：2168**

**search formula：**(((((((((((((((("Surgical Wound Infection"[Mesh]) OR (Infections, Surgical Wound[Title/Abstract])) OR (Surgical Wound Infections[Title/Abstract])) OR (Wound Infections, Surgical[Title/Abstract])) OR (Infection, Surgical Wound[Title/Abstract])) OR (Surgical Site Infection[Title/Abstract])) OR (Infection, Surgical Site[Title/Abstract])) OR (Infections, Surgical Site[Title/Abstract])) OR (Surgical Site Infections[Title/Abstract])) OR (Wound Infection, Postoperative[Title/Abstract])) OR (Wound Infection, Surgical[Title/Abstract])) OR (Infection, Postoperative Wound[Title/Abstract])) OR (Infections, Postoperative Wound[Title/Abstract])) OR (Postoperative Wound Infections[Title/Abstract])) OR (Wound Infections, Postoperative[Title/Abstract])) OR (Postoperative Wound Infection[Title/Abstract])) AND ((((((((((((((((((((("Obstetric Surgical Procedures"[Mesh]) OR (Obstetric Surgical Procedure[Title/Abstract])) OR (Procedure, Obstetric Surgical[Title/Abstract])) OR (Obstetrical Surgical Procedures[Title/Abstract])) OR (Obstetrical Surgical Procedure[Title/Abstract])) OR (Procedure, Obstetrical Surgical[Title/Abstract])) OR (Procedures, Obstetrical Surgical[Title/Abstract])) OR (Surgical Procedure, Obstetrical[Title/Abstract])) OR (Surgical Procedures, Obstetrical[Title/Abstract])) OR (Surgical Procedure, Obstetric[Title/Abstract])) OR (Surgical Procedures, Obstetric[Title/Abstract])) OR (Surgery, Obstetric[Title/Abstract])) OR (Obstetric Surgeries[Title/Abstract])) OR (Obstetric Surgery[Title/Abstract])) OR (Surgeries, Obstetric[Title/Abstract])) OR (Obstetrical Surgery[Title/Abstract])) OR (Obstetrical Surgeries[Title/Abstract])) OR (Surgeries, Obstetrical[Title/Abstract])) OR (Surgery, Obstetrical[Title/Abstract])) OR (Procedures, Obstetric Surgical[Title/Abstract])) OR (((((((((((((((((((("Gynecologic Surgical Procedures"[Mesh]) OR (Procedures, Gynecologic Surgical[Title/Abstract])) OR (Surgical Procedure, Gynecologic[Title/Abstract])) OR (Surgery, Gynecological[Title/Abstract])) OR (Gynecological Surgeries[Title/Abstract])) OR (Gynecological Surgery[Title/Abstract])) OR (Surgeries, Gynecological[Title/Abstract])) OR (Gynecologic Surgical Procedure[Title/Abstract])) OR (Surgical Procedures, Gynecologic[Title/Abstract])) OR (Gynecological Surgical Procedure[Title/Abstract])) OR (Gynecological Surgical Procedures[Title/Abstract])) OR (Procedure, Gynecological Surgical[Title/Abstract])) OR (Procedures, Gynecological Surgical[Title/Abstract])) OR (Surgical Procedure, Gynecological[Title/Abstract])) OR (Surgical Procedures, Gynecological[Title/Abstract])) OR (Procedure, Gynecologic Surgical[Title/Abstract])) OR (Gynecologic Surgery[Title/Abstract])) OR (Gynecologic Surgeries[Title/Abstract])) OR (Surgeries, Gynecologic[Title/Abstract])) OR (Surgery, Gynecologic[Title/Abstract])))

**2.Embase**

**Time：2023/5/11**

**quantities：3161**

**search formula：**

(((gynecologic AND surgical AND ('procedures'/exp OR procedures)) OR (procedures, AND gynecologic AND surgical) OR (surgical AND procedure, AND gynecologic) OR (surgery, AND gynecological) OR (gynecological AND surgeries) OR (gynecological AND surgery) OR (surgeries, AND gynecological) OR (gynecologic AND surgical AND procedure) OR (surgical AND procedures, AND gynecologic) OR (gynecological AND surgical AND procedure) OR (gynecological AND surgical AND procedures) OR (procedure, AND gynecological AND surgical) OR (procedures, AND gynecological AND surgical) OR (surgical AND procedure, AND gynecological) OR (surgical AND procedures, AND gynecological) OR (procedure, AND gynecologic AND surgical) OR (gynecologic AND surgery) OR (gynecologic AND surgeries) OR (surgeries, AND gynecologic) OR (surgery, AND gynecologic)) OR ((obstetric AND surgical AND ('procedure'/exp OR procedure)) OR (procedure, AND obstetric AND surgical) OR (obstetrical AND surgical AND procedures) OR (obstetrical AND surgical AND procedure) OR (procedure, AND obstetrical AND surgical) OR (procedures, AND obstetrical AND surgical) OR (surgical AND procedure, AND obstetrical) OR (surgical AND procedures, AND obstetrical) OR (surgical AND procedure, AND obstetric) OR (surgical AND procedures, AND obstetric) OR (surgery, AND obstetric) OR (obstetric AND surgeries) OR (obstetric AND surgery) OR (surgeries, AND obstetric) OR (obstetrical AND surgery) OR (obstetrical AND surgeries) OR (surgeries, AND obstetrical) OR (surgery, AND obstetrical) OR (procedures, AND obstetric AND surgical) OR (obstetric AND surgical AND procedure))) AND ((surgical AND wound AND infection) OR (infections, AND surgical AND wound) OR (surgical AND wound AND infections) OR (wound AND infections, AND surgical) OR (infection, AND surgical AND wound) OR (surgical AND site AND infection) OR (infection, AND surgical AND site) OR (infections, AND surgical AND site) OR (surgical AND site AND infections) OR (wound AND infection, AND postoperative) OR (wound AND infection, AND surgical) OR (infection, AND postoperative AND wound) OR (infections, AND postoperative AND wound) OR (postoperative AND wound AND infections) OR (wound AND infections, AND postoperative) OR (postoperative AND wound AND infection))

**3.Cochrane**

**time：2023/5/11**

**quantities：938**

**search formula：**

#1 MeSH descriptor: [Gynecologic Surgical Procedures] explode all trees 5658

#2 (Procedures, Gynecologic Surgical or Surgical Procedure, Gynecologic or Surgery, Gynecological or Gynecological Surgeries or Gynecological Surgery or Surgeries, Gynecological or Gynecologic Surgical Procedure or Surgical Procedures, Gynecologic or Gynecological Surgical Procedure or Gynecological Surgical Procedures or Procedure, Gynecological Surgical or Procedures, Gynecological Surgical or Surgical Procedure, Gynecological or Surgical Procedures, Gynecological or Procedure, Gynecologic Surgical or Gynecologic Surgery or Gynecologic Surgeries or Surgeries, Gynecologic or Surgery, Gynecologic):ti,ab,kw (Word variations have been searched) 7653

#3 #1 or #2 11561

#4 MeSH descriptor: [Obstetric Surgical Procedures] explode all trees 9650

#5 (Obstetric Surgical Procedure or Procedure, Obstetric Surgical or Obstetrical Surgical Procedures or Obstetrical Surgical Procedure or Procedure, Obstetrical Surgical or Procedures, Obstetrical Surgical or Surgical Procedure, Obstetrical or Surgical Procedures, Obstetrical or Surgical Procedure, Obstetric or Surgical Procedures, Obstetric or Surgery, Obstetric or Obstetric Surgeries or Obstetric Surgery or Surgeries, Obstetric or Obstetrical Surgery or Obstetrical Surgeries or Surgeries, Obstetrical or Surgery, Obstetrical or Procedures, Obstetric Surgical):ti,ab,kw (Word variations have been searched) 2753

#6 #4 or #5 11534

#7 #3 or #6 20546

#8 MeSH descriptor: [Surgical Wound Infection] explode all trees 4042

#9 (Infections, Surgical Wound or Surgical Wound Infections or Wound Infections, Surgical or Infection, Surgical Wound or Surgical Site Infection or Infection, Surgical Site or Infections, Surgical Site or Surgical Site Infections or Wound Infection, Postoperative or Wound Infection, Surgical or Infection, Postoperative Wound or Infections, Postoperative Wound or Postoperative Wound Infections or Wound Infections, Postoperative or Postoperative Wound Infection):ti,ab,kw (Word variations have been searched) 12815

#10 #8 or #9 12815

#11 #7 and #10 938

**4.WOS**

**time：2023/5/11**

**quantities：936**

**search formula：**

(TS=(Surgical Wound Infection) OR AB=(Infections, Surgical Wound OR Surgical Wound Infections OR Wound Infections, Surgical OR Infection, Surgical Wound OR Surgical Site Infection OR Infection, Surgical Site OR Infections, Surgical Site OR Surgical Site Infections OR Wound Infection, Postoperative OR Wound Infection, Surgical OR Infection, Postoperative Wound OR Infections, Postoperative Wound OR Postoperative Wound Infections OR Wound Infections, Postoperative OR Postoperative Wound Infection)) AND ((TS=(Obstetric Surgical Procedures) OR AB=(Obstetric Surgical Procedure OR Procedure, Obstetric Surgical OR Obstetrical Surgical Procedures OR Obstetrical Surgical Procedure OR Procedure, Obstetrical Surgical OR Procedures, Obstetrical Surgical OR Surgical Procedure, Obstetrical OR Surgical Procedures, Obstetrical OR Surgical Procedure, Obstetric OR Surgical Procedures, Obstetric OR Surgery, Obstetric OR Obstetric Surgeries OR Obstetric Surgery OR Surgeries, Obstetric OR Obstetrical Surgery OR Obstetrical Surgeries OR Surgeries, Obstetrical OR Surgery, Obstetrical OR Procedures, Obstetric Surgical)) OR (TS=(Gynecologic Surgical Procedures) OR AB=(Procedures, Gynecologic Surgical OR Surgical Procedure, Gynecologic OR Surgery, Gynecological OR Gynecological Surgeries OR Gynecological Surgery OR Surgeries, Gynecological OR Gynecologic Surgical Procedure OR Surgical Procedures, Gynecologic OR Gynecological Surgical Procedure OR Gynecological Surgical Procedures OR Procedure, Gynecological Surgical OR Procedures, Gynecological Surgical OR Surgical Procedure, Gynecological OR Surgical Procedures, Gynecological OR Procedure, Gynecologic Surgical OR Gynecologic Surgery OR Gynecologic Surgeries OR Surgeries, Gynecologic OR Surgery, Gynecologic)))

**5.VIP**

**time：2023/5/11**

**quantities：366**

**search formula：**

妇产科 or 妇科 or 产科 or 妇科外科手术 or 产科外科手术 or 妇科手术 or 产科手术

外科伤口感染 or 切口感染 or 手术切口感染

**6.CNKI**

**time：2023/5/12**

**quantities：794**

**search formula：**

**妇科手术 + 妇科外科手术**

**产科手术 + 产科外科手术**

**妇产科 + 临床妇产科**

**切口感染 + 手术部位感染 + 手术切口感染**

**7.万方**

**time：2023/5/12**

**quantities：968**

**search formula：**

((主题:(妇科手术) or 题名或关键词:(妇科外科手术 or 妇科)) or (主题:(产科手术) or 题名或关键词:(产科外科手术 or 产科)) or (主题:(妇产科) or 题名或关键词:(临床妇产科))) and (主题:(外科伤口感染) or 题名或关键词:(切口感染 or 手术切口感染))

**8.CBM**

**time：2023/5/12**

**quantities：2098**

**search formula：**

(( "外科伤口感染"[常用字段:智能] OR "手术部位感染"[常用字段:智能])) AND (( "妇科外科手术"[常用字段:智能] OR "妇外科"[常用字段:智能] OR "妇科手术"[常用字段:智能]) OR( "产科外科手术"[常用字段:智能] OR "产科手术"[常用字段:智能]) OR( "妇产科"[常用字段:智能] OR "临床妇产科"[常用字段:智能]))

11429
